# Demonstration of biological activities of extracts from *Isodon rugosus* Wall. Ex Benth: Separation and identification of bioactive phytoconstituents by GC-MS analysis in the ethyl acetate extract

**DOI:** 10.1186/s12906-017-1798-9

**Published:** 2017-05-30

**Authors:** Anwar Zeb, Farhat Ullah, Muhammad Ayaz, Sajjad Ahmad, Abdul Sadiq

**Affiliations:** Department of Pharmacy, University of Malakand, Chakdara, Dir, KPK (L)18000 Pakistan

**Keywords:** Antibacterial, MICs, Anthelmintic, *Ascaridia galli*, Anti-termites, Anti-Pharaoh, *Isodon rugosus*, GC-ms

## Abstract

**Background:**

Since long, natural sources have been explored for possible managements of various diseases. In this context, the study is designed to evaluate *Isodon rugosus* Wall. ex Benth for biological potentials including antibacterial, anthelmintic, insecticidal, anti-termites and anti-Pharaoh activities followed by GC-MS analysis of active fraction to identify various bioactive compounds.

**Methods:**

*I. rugosus* was investigated against eight bacterial strains using well diffusion method and microdilution method with ceftriaxone as positive control. Similarly, the insecticidal activity was carried out against *Tribolium castaneum*, *Rhyzopertha dominica*, *Monomorium pharaonis* and *Heterotermis indicola* following contact toxicity method. Likewise, anthelmintic activity was performed against *Ascaridia galli* and *Pherethima posthuma* using albendazole as positive control, in which the paralysis and death times of the worms were observed. The GC-MS analysis of the most active solvent fraction was performed for identifications of various bioactive compounds.

**Results:**

Among the tested samples of *I. rugosus*, flavonoids and ethyl acetate fraction exhibited high antibacterial activities. The crude saponins showed highest anthelmintic activity against *Pherethima posthuma* and *Ascaridia galli* with death times of 27.67 and 29.22 min respectively at concentrations of 40 mg/ml. In insecticidal activity, chloroform fraction and saponins exhibited notable results against *R. dominica* (60 and 70%) and *T. castaneum* (70 and 76%) at concentration of 200 mg/ml. In anti-termite assay, all the plant samples showed overwhelming results, i.e. all the 25 termites were killed on the 3rd day. Similarly, in anti-Pharaoh activity, the chloroform, ethyl acetate and saponins fractions were most potent, each exhibiting LD_50_ of <0.1 mg/ml. In GC-MS analysis, total of 57 compounds were identified. Some of the bioactive compounds identified in GC-MS analysis are palmitic acid, hinokiol, α-amyrin, phytol, ethyl linolate, cyclohexanone, hinokione, methyl palmitate, ethyl palmitate and stigmasterol acetate.

**Conclusions:**

Based on our current results, it can be concluded that *I. rugosus* possess strong antibacterial, insecticidal and anthelmintic potentials having crude saponins and ethyl acetate as the most active fractions. The GC-MS analysis and biological assays reveal that ethyl acetate fraction is a suitable target for the isolation of diverse array of bioactive compounds.

## Background

Infectious diseases are among the leading health problems, accounting for 41% of global disease burden [1]. Diseases caused by bacteria and parasites are still challenging to public health. Their impact is particularly large in developing countries due to unavailability of relevant medicines and the emergence of widespread drug resistance [2–4]. The clinical efficacy of many existing antibiotics has been endangered by the emergence of MDR pathogens [5, 6]. To triumph over MDR pathogens, it is important to develop novel and more effective antimicrobial agents, especially with a different mechanism of action [7]. It is obvious that despite synthetic compounds, natural raw materials are the key sources for novel therapeutic agents for the management of various ailments like infectious diseases [8, 9].

Helminthiasis is among the most widespread parasitic infections in humans, distressing an enormous population of the world. Approximately two billions people are suffering from parasitic worms infections [10]. Majority of helminths mediated infections are limited to tropical regions and cause huge hazard to health leading to undernourishment, anaemia, eosinophilia, physical, mental sluggishness and pneumonia [11]. Parasitic infections also cause lymphatic filariasis leading to elephantiasis, onchocerciasis called river blindness and schistosomiasis. These infections are more common in populations living in endemic areas with poor hygiene and socio-economic problems [12]. Livestock and crops are also affected by parasitic worms thus leading to reduction in crops and milk production [13]. There is a substantial improvement in helminths control but due to emergence of anthelmintic resistance to currently available drugs. So, it is important to search alternative strategies against gastrointestinal nematodes [14]. Similarly, most of the available insecticidal from chemical sources are hazardous to human health and are associated with environmental risks [15]. Therefore, alternative resources must be explored to substitute the chemical pesticides. Pesticides from natural sources are advantageous as they are selective in targeting specific species and also have unique modes of action [16].

*Isodon rugosus* Wall. ex Benth. belongs to the family Lamiaceae. *Isodon*, an important genus of this family is a rich source containing a large number of bioactive compounds. The *Isodon* species have been used as anti-tumor, anticancer, antimicrobial, insecticide and anthelmintic [17, 18]. *Isodon rugosus* has been used ethnomedicinally as an antimicrobial and anthelmintic [19, 20]. This plant has also been used traditionally against skin infections and for the treatment of scabies [21, 22]. This specie has also been verified scientifically to possess variety of pharmacological activities [23]. Based on the published literature, the current study is designed to evaluate various fractions of *I. rugosus* for the antimicrobial, anthelmintic and insecticidal potentials along with identifications and sorting out of bioactive compounds by GC-MS analysis.

## Methods

### Plant collection and extraction

The plant was collected from Dir (KPK), Pakistan in the month of June and was identified by plant taxonomist at Department of Botany, Shaheed Benazir Bhuto University Dir (KPK), Pakistan. The plant sample was kept at the herbarium of the same university with voucher number 1016AZ. Fresh aerial parts (11 kg) were separated from the rest of the plant and rinsed with clean water to remove any dust particles followed by shade drying for 23 days. The dried plant parts were cut into small pieces and grinded into coarse powder with the help of a grinder. The powdered material (6 kg) was macerated in 80% methanol (25 L) for 18 days and the process was repeated three times. The filtrates were combined and concentrated under reduced pressure using rotary evaporator [24, 25]. A brown semisolid mass (380 g) of the crude methanolic extracts was obtained.

### Fractionation

The crude methanolic extract was transferred into a separating funnel and diluted with 500 ml of distilled water. *n*-Hexane (500 ml) was added to it with vigorous shaking and kept for a while to form two layers. The *n*-hexane layer was separated and repeated the same procedure three times by adding 500 ml of *n*-hexane each time. All the *n*-hexane layers were combined and concentrated at reduced pressure using rotary evaporator. The final concentrated weight of *n*-hexane fraction was 19 g. In a similar way like *n*-hexane fraction, the chloroform and ethyl acetate fractions were extracted sequentially weighing 27 and 80 g respectively. The dry water residue (aqueous fraction) was collected at the end weighing 125 g [26, 27].

### Extraction of crude saponins

The crude saponins from *I. rugosus* were obtained by adding 20 g of the plant powder in a conical flask having 100 ml of 20% ethanol. The sample was heated in water bath for 4 h at 55 °C with continuous stirring. The mixture was filtered and extracted again with 200 ml of 20% ethanol. The volume was heated up to 60 °C in water bath and got 40 ml of greenish color residue. The residue was added into the separating funnel followed by the addition of diethyl ether (20 ml) with vigorous shaking. After vigorous shaking, the separating funnel was put in a stand to get two distinct layers. The upper diethyl ether layer was discarded while the lower aqueous layer obtained was diluted with *n*-butanol (60 ml). This extract was washed with 10 ml of 5% sodium chloride solution. The solution was evaporated by keeping in water bath to obtained the dried saponins [26].

### Extraction of Flavonoids

Plant sample having weight of 20 g was heated at 50 °C in 200 ml of HCl (2 M) under reflux for 30 min. The plant sample was filtered using Whatman No. 42 filter paper after getting cold. The filtrate obtained was poured into a separating funnel and added equal volume of ethyl acetate. The total amount of flavonoids present in the plant sample was precipitated, which were collected from the sample using a filter paper. The weight of flavonoids was 1.4 g (7%) [28].

### Gas chromatography (GC) analysis

Ethyl acetate fraction was subjected to GC analysis and the GCMS was done by means of an Agilent USB-393752 gas chromatograph (Agilent Technologies, Palo Alto, CA, USA) with HHP-5MS phenylmethylsiloxane 5% with capillary column (30 m × 0.25 mm × 0.25 μm; Restek, Bellefonte, PA) equipped with an FID detector. Initially, the temperature of oven was maintained at 70 °C for 1 min, and then increased the required temperature slowly and step wise to 180 °C at the rate of 6 °C/min for 5 min, and at the final stage increased the temperature to 280 °C at the rate of 5 °C/min for 20 min. The temperatures of the Injector and detector were set at 220 °C and 290 °C, respectively. Helium was used as a carrier gas with a flow rate of 1 ml/min [29].

### Gas chromatography–mass spectrometry (GC/MS) analysis

Ethyl acetate fraction was subjected to GC/MS analysis and the processed was done by means of an Agilent USB-393752 gas chromatograph (Agilent Technologies, Palo Alto, CA, USA) with a HHP-5MS phenylmethylsiloxane 5% with a capillary column (30 m × 0.25 mm × 0.25 μm film thickness; Restek, Bellefonte, PA) prepared with an Agilent HP-5973 mass selective detector in the electron impact mode (ionization energy: 70 ev) working under similar experimental background as illustrated for GC.

### Identification of components

Identification of the compounds present in the ethyl acetate fraction of *I. rugosus* was carried out on the base of comparison of their relative retention indices of each component with those of the authentic compounds present in the literature. Further identifications of the compounds were carried out from the spectral data obtained from the Wiley and NIST libraries and additional identifications were carried out by completed comparisons of the fragmentation pattern of the mass spectra with the reported in literature [30].

### Antibacterial investigations

#### Bacterial strains

Eight bacterial strains including *Escherichia coli* (739), *Klebsiella pneumoniae* (700603), *Pseudomonas aeruginosa* (27853), *Enterococcus faecalis* (29212), *Proteus mirabilis* (13315), *Staphylococcus aureus* (29213), *Bacillus cereus* and *Salmonella typhi* were used to investigate antibacterial potential of plant’s samples. The samples were provided by the Microbiology Laboratory of Department of Microbiology, Quaid-e-Azam University, Islamabad. Bacteria were preserved in freeze-dried condition at 4 °C in stab slant agar until later use.

### Preparation and standardization of inoculums

Bacterial cultures were grown for 24 h at 37 °C and suspensions with cell density of 1 × 10^8^ CFU/ml, were prepared using McFarland standard and was further diluted to a cell density of 1 × 10^6^ CFU/ml using a UV visible spectrophotometer (Thermo electron corporation USA) at 625 nm and the standardization was maintained for the period of the study [31].

### Antibacterial assay

Preliminary antibacterial activity of plant extracts were investigated using well assay technique [32, 33]. Briefly, Nutrient agar plates were prepared and inoculated with the test organisms aseptically under laminar flow hood and were properly labeled. Wells of 5 mm diameter were made in the plates using sterile cork borer. Samples were prepared having concentrations of 10 mg/ml. From each sample, 100 μl of extract was added into each well using micropipette. The standard drug ceftriaxone was used as positive control, which was added into the central well contained in nutrient agar media in each Petri plate. After inoculation and addition of the samples, the Petri plates were kept in incubator at 37 °C for 24 h. Diameter of inhibitory zones were determined around each bore after 24 h and were compared with positive control. Experiments were performed in triplicate and the data obtained was represented as mean ± SEM.

### Determination of MICs

MICs were determined using microdilution method as described by National Committee for Clinical Laboratory Standards (NCCLS) [34]. Briefly, the plant’s samples were prepared at concentrations of 50 mg/ml (stock solution) in sterile distilled water and serially diluted with distilled water to 20, 17.5, 15, 12.5, 10, 7.5, 5, 2.5, 1 and 0.5 μg/ml using nutrient broth followed by inoculation with 0.2 ml suspension of the test organisms. After 24 h of incubation at 37 °C, the test tubes were visually observed for growth. The lowest concentration at which no growth of the microorganism was observed was considered as MIC of the plant extract against the relevant strains of the microorganisms.

### Anthelmintic investigations

For the anthelmintic activity of *I. rugosus,* adult earthworms (*Pherethima posthuma*) and roundworms (*Ascaridia galli*) were used. Earthworms having average length of 7–8 cm and width 0.1–0.2 cm were collected from the muddy soil near Department of Pharmacy, University of Malakand, KPK, Pakistan. The selection of earthworms (*P. posthuma*) is based on close physiologic and anatomic resemblance with the human intestinal round worm parasites *Ascaris lumbricoides* [35]. Roundworms were collected from the intestines of chicken. The chickens were slaughtered, their intestines were dissected after the removal of fecal material with the help of normal saline and roundworms were collected from their intestines. These roundworms (*A. galli*) have close resemblance with that of the roundworm parasite (*Ascaris lumbricoides*) found in the human intestine. Both types of worms were divided into different groups, each group containing six (6) worms. All the extracts, their fractions, crude saponins and albendazole solutions were prepared in 10, 20 and 40 mg/ml. From each solution, 25 ml of the sample was transferred to sterilized Petri dishes (150 × 15 mm) followed by addition of earthworms using forceps. Observations were made for the time taken when the worms lost their motility as paralysis time and death was concluded when no movement was observed even with vigorous shaking in hot water at 50 °C. Both paralysis and death times were recorded for different fractions and were compared with the positive control [36].

### Insecticidal investigations

#### Anti-beetle

The anti-beetle activity was carried out against *R. dominica* (grain borer) and *T. castaneum* (flour beetle) following the procedure previously described [37]. Various dilutions of extracts were prepared in methanol, i.e., 200, 100 and 50 mg/ml and filter paper was wetted with the 5 ml of each test sample solution in Petri dishes and kept overnight to evaporate the solvent. After evaporation of the solvents, 20 healthy insects were shifted to each Petri dish and kept for 24 h at room temperature with 50% of relative humidity. After 24 h, the results were checked by counting the number of dead and alive insects. Permethrin was used as positive control while the volatile solvents were used as negative control.

#### Anti-termite investigations

The anti-termite activity of crude saponins, methanolic extracts and resultant fractions of *I. rugosus* was conducted following standard procedure [38]. In this procedure the plant’s samples were assayed against *Heterotermes indicola* (termites). Sterilized filter papers were kept in Petri dishes equal to the size of the Petri dishes. Samples were prepared by dissolving 2 mg/ml of each extract of the plant in respective solvents. The filter papers were kept for 24 h so that the solvents become fully evaporated from it. Each filter paper was kept in the labeled Petri dishes. In each Petri dish, 25 termites were placed and were kept for 24 h at room temperature. After 24 h, the number of dead and alive termites was counted. Similarly, the results were also recorded on the second and third day. This activity was performed in triplicate and the average termites killed each day were recorded. Permethrin was used as positive control. The LD_50_ of each sample was calculated by graph prism pad software and Microsoft Excel.

#### Anti-Pharaoh (ants) investigations

The anti-Pharaoh activity was performed by the direct contact toxicity method described by Ahn et al. [37]. From each fraction, solutions of 0.5, 1, 2, 4, 6 and 8 mg/ml concentrations were prepared. Each concentration was added into Petri dish containing sterilized filter paper and kept for 12–15 h for evaporation of the solvent. After evaporation, 20 Pharaoh ants were added into each Petri dish. The Petri dishes containing distilled water treated filter paper served as a control. The Petri dishes were placed at room temperature for 24 h and numbers of dead and alive ants were observed in each Petri dish. Permethrin was used as positive control. The percent mortality of each sample was calculated and was compared with the positive control.

### Statistical analysis

Each experiment was performed in three replicates and values were expressed as mean ± SEM. two way ANOVA followed by multiple comparison Bonferroni’s test. The *P* values less than 0.05 were considered as statistically significant. LD_50_ values were calculated by a linear regression analysis among the percent inhibition against the extract concentrations via Excel program and GraphPad Prism software.

## Results

### Gas chromatography mass spectroscopy

**Fig. 1:**
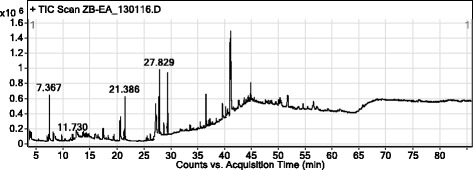
Chromatogram of Ethyl acetate fraction of *Isodon rugosus*

**Fig. 2:**
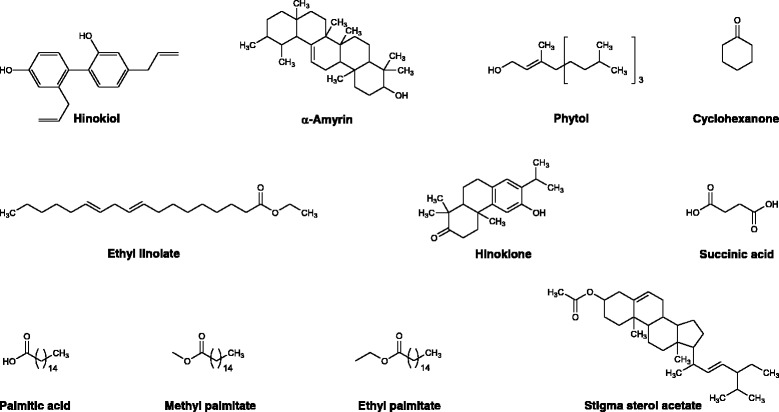
Bioactive compounds identified in the ethyl acetate fraction of *Isodon rugosus*

**Fig. 3:**
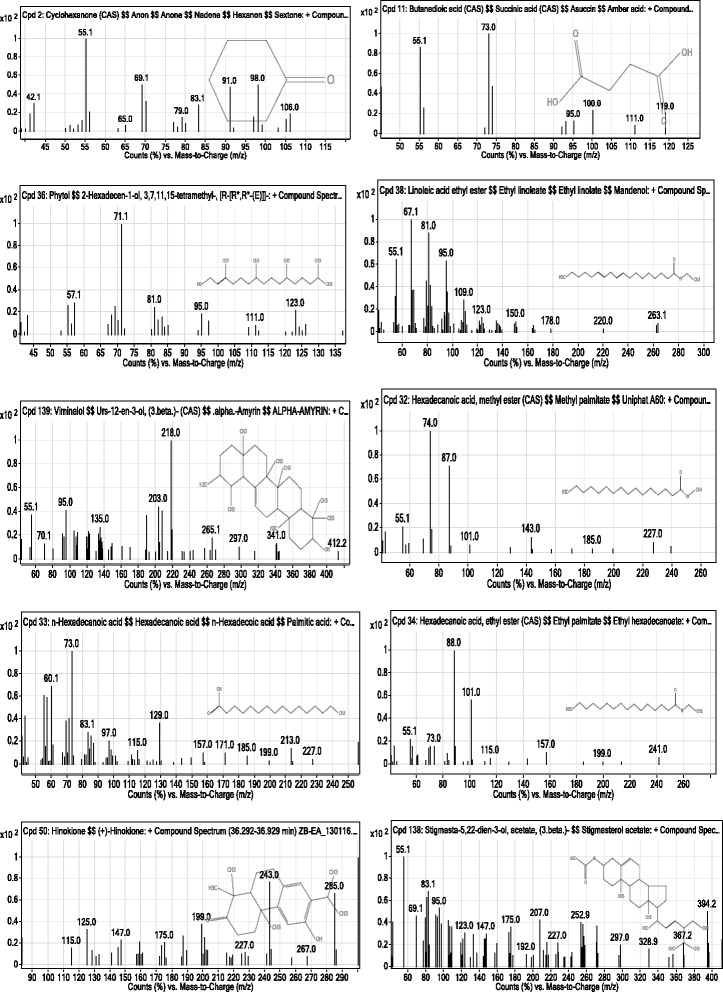
Mass spectra of bioactive compounds in ethyl acetate fraction of *Isodon rugosus*

The GC-MS analysis of ethyl acetate fraction revealed the identifications of 57 compounds which have been enlisted in Table 1. Various parameters of major compounds in this fractions i.e., retention time, percent area, base peak, width etc. has also been summarized in Table 2. The GC-MS chromatogram of ethyl acetate fraction (Fig. 1) showed the peak areas for various compounds including the bioactive compounds. The structures of the bioactive compounds are shown in Fig. 2. The integration pattern of dominant compounds determined by GC-MS analysis is provided in Fig. 3.

**Table 1:** List of all compounds identified in the GC-MS analysis of ethyl acetate fraction of *Isodon rugosus*

S. No	Compound Label	RT	Name	Formula	Hits (DB)
1.	Cyclohexanone (CAS) $$ Anon $$ Anone $$ Nadone $$ Hexanon $$ Sextone	3.858	Cyclohexanone	C6H10O	10
2.	Ethanol, 2-ethoxy-, acetate (CAS) $$ 2-Ethoxyethyl acetate $$ Oxitol acetate	3.936	Oxitol acetate	C6H12O3	10
3.	Cyclopentane, 1,1,3,4-tetramethyl-, trans- (CAS)	4.726	Nf	C9H18	10
4.	1-(4-Tosyloxybutyl)-3-(1-methoxy-1-methylethoxy)clopentene	4.842	Nf	C20H30O5S	1
5.	(1.alpha.,5.alpha.,6.alpha.,7.alpha.)-6,7-(Z,E)-Dipropenyl-3-oxabicyclo[3.2....	5.428	Nf	C12H18O	6
6.	Butanedioic acid, ethyl methyl ester (CAS) $$ 1-Ethyl 4-methyl succinate	7.031	Nf	C7H12O4	10
7.	N-Butylpropargylamine	7.156	Butylpropargylamine	C7H13N	10
8.	2-Cyclohexen-1-one, 3,5,5-trimethyl- (CAS) $$ 3,5,5-Trimethyl-2-cyclohexenone	7.366	Nf	C9H14O	10
9.	Succinic acid, butyl ethyl ester	8.151	Nf	C10H18O4	10
10.	Butanedioic acid (CAS) $$ Succinic acid (CAS) $$ Asuccin $$ Amber acid	8.234	Amber acid	C4H6O4	10
11.	Undecane, 4-methyl- (CAS) $$ 4-Methylundecane $$ 4 - methyl - undecane	8.456	Nf	C12H26	10
12.	1-(1,1-dimethyl-2-propenyl)-1-cyclohexene	10.256	Nf	C11H18	10
13.	Dodecane, 2-methyl- (CAS) $$ 2-Methyldodecane $$ 11-Methyldodecane	11.258	Nf	C13H28	10
14.	(+)-(E)-N,N-Di-tert-butoxycarbonyl-1,3-diphenylprop-2-enyamine	12.008	Nf	C25H31NO4	1
15.	Benzene, 1-(bromomethyl)-3-nitro- $$ m-(Bromomethyl)nitrobenzene	12.284	Nf	C7H6BrNO2	10
16.	rac-.gamma.(2)-muurolene	12.418	rac-γ(2)-muurolene	C16H24	10
17.	Decane, 3-bromo- $$ 3-Bromodecane #	12.54	Nf	C10H21Br	1
18.	Acetic Acid 2-(4-Hydroxyphenyl)ethyl Ester	13.523	Nf	C10H12O3	10
19.	Tetradecane, 1-chloro- $$ Myristyl chloride $$ Tetradecyl chloride	13.738	Myristyl chloride	C14H29Cl	10
20.	alpha.-Amorphene $$. ALPHA. AMORPHENE $$ 6.alpha.-Cadina-4,9-diene, (−)-	14.153	α-Amorphene	C15H24	10
21.	alpha.-Copaene $$ Copaene (CAS) $$ Copaen $$ (−)-.alpha.-Copaene	14.516	Copaen	C15H24	10
22.	alpha.-Cadinol $$ Cadin-4-en-10-ol $$ l-.alpha.-Cadinol $$ 10-.ALPHA.-CADINOL	14.64	α-Cadinol	C15H26O	10
23.	4-((1E)-3-Hydroxy-1-propenyl)-2-methoxyphenol	15.859	Nf	C10H12O3	10
24.	(−)-Loliolide $$ Loliolide $$ Loliolid $$ Digiprolactone $$ Calendin	16.428	Loliolide	C11H16O3	2
25.	Neophytadiene $$ 7,11,15-TRIMETHYL,3-METHYLENE-1-HEXADECENE	17.314	Neophytadiene	C20H38	10
26.	R-7-endo-p-phenylbenzoyloxy-6-anti-(.beta.-phenylethenyl)-2-oxa-bicyclo[3.3....	17.447	Nf	C27H24O3	1
27.	2-Hexadecen-1-ol, 3,7,11,15-tetramethyl-, [R-[R*,R*-(E)]]- (CAS)	18.25	Nf	C20H40O	10
28.	Hexadecanoic acid, methyl ester (CAS) $$ Methyl palmitate $$ Uniphat A60	19.316	Methyl palmitate	C17H34O2	10
29.	n-Hexadecanoic acid $$ Hexadecanoic acid $$ n-Hexadecoic acid $$ Palmitic acid	20.498	Palmitic acid	C16H32O2	10
30.	Hexadecanoic acid, ethyl ester (CAS) $$ Ethyl palmitate $$ Ethyl hexadecanoate	21.386	Ethyl palmitate	C18H36O2	10
31.	CIS-11,14,17-EICOSATRIENOIC ACID ME	25.548	Nf	C21H36O2	10
32.	Phytol $$ 2-Hexadecen-1-ol, 3,7,11,15-tetramethyl-, [R-[R*,R*-(E)]]-	26.041	Phytol	C20H40O	10
33.	17-Octadecynoic acid	27.132	Nf	C18H32O2	10
34.	Linoleic acid ethyl ester $$ Ethyl linoleate $$ Ethyl linolate $$ Mandenol	27.616	Mandenol	C20H36O2	10
35.	Acetic acid, chloro-, hexadecyl ester $$ Chloro-acetic acid hexadecyl ester	27.817	Nf	C18H35ClO2	10
36.	9,12,15-Octadecatrienoic acid, methyl ester, (Z,Z,Z)- $$ Methyl linolenate	27.831	Methyl linolenate	C19H32O2	10
37.	Octadecanoic acid, ethyl ester (CAS) $$ Ethyl stearate $$ Ethyl octadecanoate	28.617	Ethyl stearate	C20H40O2	10
38.	Neophytadiene $$ 7,11,15-TRIMETHYL,3-METHYLENE-1-HEXADECENE	29.321	Neophytadiene	C20H38	10
39.	Hexadecanoic acid, ethyl ester (CAS) $$ Ethyl palmitate $$ Ethyl hexadecanoate	33.473	Ethyl palmitate	C18H36O2	10
40.	Naphthalene, 1,2,3,4-tetrahydro-1-isopropyl-1,2,4,4,7-pentamethyl-	35.4	Nf	C18H28	1
41.	3,4,8-trimethyl-9-oxy-1-trimethylsilyloxybicyclo[4.3.1]non-1-ene	35.883	Nf	C15H26O2Si	10
42.	Di-(2-ethylhexyl)phthalate $$ 1,2-BENZENEDICARBOXYLIC ACID	36.417	Nf	C24H38O4	10
43.	Hinokione $$ (+)-Hinokione	36.604	Hinokione	C20H28O2	10
44.	[1, 3, 4]thiadiazolo[3,2-a]pyrimidin-7-one, 6-(2,5-dimethoxybenzylidene)-5-imi...	37.056	Nf	C15H14N4O3S	1
45.	Hinokiol $$ (+)-Hinokiol	37.242	Hinokiol	C20H30O2	4
46.	7,8,9-Trimethoxy-4,5-dihydro-1H-benzo[g]indazole	39.446	Nf	C14H16N2O3	1
47.	13b–methyl-3-propyl-1,3,4,6,7,8,13,13b–octahydro-2 h-pyrido[1′,2′:1,2]azepino...	40.14	Nf	C20H28N2	10
48.	7,7-Dimethyl-6,7-diihydro-5H-benzo[f]pyrano[2,3-h]quinoxaline	40.607	Nf	C17H16N2O	2
49.	3-[(3,4-dimethoxy-benzylamino)-methyl]-8a–methyl-5-methylene-decahydro-napht...	40.934	Nf	C24H33NO4	1
50.	1-Hexadecanol (CAS) $$ Cetal $$ Ethal $$ Ethol $$ Cetanol $$ Cetylol $$ Adol 52	43.494	Cetylol	C16H34O	10
51.	dl-.alpha.-Tocopherol $$ (.+/−.)-.alpha.-Tocopherol $$ Vitamin E	45.326	Vitamin E	C29H50O2	7
52.	1,3-Diethyladamantane	49.926	Nf	C14H24	2
53.	Stigmasta-5,22-dien-3-ol, acetate, (3.beta.)- $$ Stigmasterol acetate	50.163	Stigmasterol acetate	C31H50O2	6
54.	Viminalol $$ Urs-12-en-3-ol, (3.beta.)- (CAS) $$ .alpha.-Amyrin $$ ALPHA-AMYRIN	50.299	Viminalol	C30H50O	10
55.	4,6-Dimethoxy-2,3-diphenyl-7-(1-pyrrolin-2-yl)indole	51.53	Nf	C26H24N2O2	3
56.	Stigmast-4-en-3-one $$ 4-Stigmasten-3-one $$ Sitostenone	51.622	Sitostenone	C29H48O	1
57.	4-Hydroxy-1,2-dimethoxyanthraquinone	62.475	Nf	C16H12O5	3

**Table 2:** Various parameters major compounds in GC-MS of ethyl acetate fraction of *Isodon rugosus*

S.No	RT	Height	Height %	Area	Area %	Area Sum %	Base Peak m/z	Width
1.	3.857	54,336	6.81	66,940	1.91	0.65	55.1	0.05
2.	3.938	57,317	7.19	70,955	2.03	0.69	43.1	0.054
3.	7.029	40,590	5.09	80,194	2.29	0.78	115	0.07
4.	7.367	581,648	72.95	1E + 06	29.17	9.92	82	0.107
5.	8.15	74,641	9.36	122,166	3.49	1.19	101	0.054
6.	9.698	44,503	5.58	70,074	2	0.68	70.1	0.06
7.	11.73	62,937	7.89	90,543	2.59	0.88	93	0.05
8.	21.386	542,089	67.99	2E + 06	66.56	22.64	88	0.171
9.	27.829	797,352	100	3E + 06	100	34.02	79	0.178
10.	29.319	754,398	94.61	3E + 06	78.36	26.66	68.1	0.137

### Antibacterial studies

#### Sensitivity pattern

The bacterial strains were exposed to different antibiotic groups to determine their susceptibility pattern as shown in Table 3. All strains were found resistant to more than three antibiotics and therefore were termed as multi drug resistant (MDR) strains.

**Table 3:** Antibiotic Susceptibility pattern of selected bacterial strains to different antibiotic groups

Bacterial strains	Diameter of the inhibitory zone (mm) Mean ± SEM (*n* = 5)					
	Ciprofloxacin	Cefixime	Amoxycillin	Gentamycin	Cephradine	Cloxacillin
*Escherichia coli* (739)	24.67 ± 1.20	18.33 ± 0.88	07.67 ± 0.67	26.67 ± 0.67	06.67 ± 0.88	09.33 ± 0.33
*Klebsiella pneumoniae* (700603)	19.00 ± 0.57	06.67 ± 0.67	08.00 ± 0.57	22.00 ± 0.57	08.67 ± 0.88	06.67 ± 1.20
*Pseudomonas aeruginosa* (27853)	22.33 ± 0.88	16.67 ± 1.20	07.67 ± 1.20	20.67 ± 1.20	10.33 ± 1.45	08.33 ± 0.33
*Enterococcus faecalis* (29212)	16.33 ± 0.33	12.33 ± 0.88	09.33 ± 0.88	26.67 ± 0.67	14.00 ± 1.15	06.67 ± 0.67
*Proteus mirabilis* (13315)	18.67 ± 1.20	06.67 ± 0.33	10.67 ± 0.33	27.33 ± 0.88	10.00 ± 0.57	12.33 ± 0.88
*Staphylococcus aureus* (29213)	26.67 ± 0.67	08.33 ± 0.33	14.00 ± 0.57	29.00 ± 0.57	12.33 ± 0.88	08.00 ± 0.57
*Bacillus cereus*	23.00 ± 0.57	11.67 ± 0.67	11.33 ± 0.88	26.00 ± 1.15	12.67 ± 0.67	09.67 ± 1.20
*Salmonella typhi*	23.33 ± 0.33	26.67 ± 1.20	7.67 ± 1.20	24.33 ± 0.33	10.00 ± 1.15	07.00 ± 1.15

### Zone of inhibitions

Results of antibacterial activity (ZOI) are shown in Table 4. Among all the tested samples, flavonoids were most active against all the strains and its antibacterial potential was comparable to the standard drug ceftriaxone. Among other fractions, ethyl acetate was most effective against *S. aureus* and *S. typhi* displaying inhibitory zones of 29.3 ± 0.28 and 28.2 ± 0.35 mm respectively. Crude saponins were also effective against *S. aureus, E. coli, B. cereus* and *K. pneumoniae* causing inhibitory zones of 27.2 ± 0.23, 24.1 ± 0.52, 26.3 ± 0.34 and 21.3 ± 0.39 mm respectively. The crude methanolic extract and chloroform fraction were inactive against *B. cereus*, whereas the aqueous fraction was inactive against *E. coli* and *E. faecalis*. *n*-Hexane fraction was also inactive against *E. coli*. All the test samples were approximately least active against *E. coli*.

**Table 4:** Antibacterial activity of *Isodon rugosus* against various bacterial strains expressed in mm as ZOI

Samples	*P. m*	*S. a*	*E. c*	*B. c*	*S. t*	*K. p*	*P. a*	*E. f*
Ir.Cr	17.2 ± 0.37	9.1 ± 0.34	7.2 ± 0 .55	---	28.2 ± 0.40	18.2 ± 0.35	9.3 ± 0.31	21.3 ± 0.36
Ir.Hex	07.3 ± 0.34	9.1 ± 0.52	---	7.1 ± 0.49	18.2 ± 0.37	17.2 ± 0.37	8.3 ± 0.25	23.3 ± 0.36
Ir.Cf	11.2 ± 0.32	19.2 ± 0.47	7.1 ± 0.26	---	6.1 ± 0.46	15.3 ± 0.35	9.2 ± 0.31	19.3 ± 0.39
Ir.EtAc	9.2 ± 0.40	29.3 ± 0.28	21.2 ± 0.40	17.1 ± 0.46	28.2 ± 0.35	18.2 ± 0.40	7.1 ± 0.46	8.2 ± 0.37
Ir.Aq	21.2 ± 0.31	9.2 ± 0.31	---	13.2 ± 0.20	9.2 ± 0.40	9.2 ± 0.40	17.2 ± 0.37	---
Ir.Sp	18.2 ± 0.43	27.2 ± 0.23	24.1 ± 0.52	26.3 ± 0.34	8.2 ± 0.37	21.3 ± 0.39	9.2 ± 0.40	19.3 ± 0.38
Ir.Flv	33.1 ± 0.49	16.2 ± 0.54	12.2 ± 0.54	31.3 ± 0.31	16.2 ± 0.23	27.3 ± 0.35	19.2 ± 0.38	13.2 ± 0.45
Ceft	35.2 ± 0.37	31.1 ± 0.34	9.2 ± 0.44	29.3 ± 0.42	19.2 ± 0.37	25.4 ± 0.40	36.1 ± 0.34	21.3 ± 0.41

Results are expressed as zone of inhibition (ZOI). Data is represented as mean ± SEM, (*n* = 3).

Keys:

*P. m Proteus mirabilis, S. a Streptococcus aureus, E. c Escherichia coli, B. c Bacillus cereus, S. t Salmonella typhi, K. p Klebsiella pneumonia, P. a Pseudomonas aeruginosa, E. f Enterococcus faecalis, Ir.Cr* Crude methanolic extract, *Ir.Hex n*-hexane fraction, *Ir.*Cf Chloroform fraction, *Ir.EtAc* Ethyl acetate fraction, *Ir.Aq* Aqueous fraction, *Ir.Sp* Saponins, *Ir.Fl* Flavonoids, *Ceft* Ceftriaxone

### Minimum inhibitory concentration (MICs)

Among all the tested samples, crude methanolic extract was found most effective against *S. aureus* and *B. cereus* with minimum inhibitory concentrations (MICs) of 2.50 ± 0.00 and 2.66 ± 1.30 μg/ml. MIC of *n*-hexane fraction against *B. cereus was* 1.33 ± 0.60 μg/ml which was comparable to positive control ceftriaxone displaying MIC of 0.83 ± 0.16 μg/ml. The chloroform fraction was most effective against *K. pneumonia* and *E. coli,* whose visible growth was inhibited at 1.16 ± 0.66 and 1.00 ± 0.00 μg/ml respectively. Saponins fraction was most potent against *P. mirabilis, S. aureus* and *K. pneumonia* with MICs of 1.00 ± 0.00, 0.83 ± 0.16 and 1.00 ± 0.00 μg/ml respectively. Flavonoids were also found equally effective against the tested microbes. All the results of minimum inhibitory concentrations (MICs) are summarized in Table 5.

**Table 5:** Minimum inhibitory concentrations for different fractions of *Isodon rugosus* against the tested bacterial strains expressed as μg/ml

*Samples*	*P. m*	*S. a*	*E. c*	*B. c*	*S. t*	*K. p*	*P. a*
Ir.Cr	6.66 ± 0.83	2.50 ± 0.00	8.33 ± 0.83	2.66 ± 1.30	12.50 ± 2.88	8.33 ± 0.83	4.16 ± 1.66
Ir.Hex	18.33 ± 0.83	16.66 ± 0.83	2.83 ± 1.16	1.33 ± 0.60	8.33 ± 2.20	10.83 ± 2.20	12.50 ± 1.44
Ir.Cf	2.50 ± 0.00	3.66 ± 1.96	1.00 ± 0.00	5.33 ± 2.60	2.01 ± 0.50	1.16 ± 0.66	2.50 ± 0.00
Ir.EtAc	7.50 ± 1.44	7.50 ± 1.44	4.16 ± 0.83	2.83 ± 1.16	3.33 ± 0.83	1.33 ± 0.60	2.83 ± 1.16
Ir.Aq	9.16 ± 0.83	11.66 ± 0.83	10.00 ± 1.44	10.83 ± 2.20	7.50 ± 1.44	5.00 ± 1.44	5.00 ± 1.44
Ir.Sp	1.00 ± 0.00	0.83 ± 0.16	5.00 ± 1.44	2.50 ± 0.00	4.50 ± 1.89	1.00 ± 0.00	3.33 ± 0.83
Ir.Flv	2.83 ± 1.16	1.00 ± 0.00	1.50 ± 0.50	6.66 ± 2.20	2.00 ± 0.50	2.66 ± 1.30	5.00 ± 1.44
Ceftriaxone	0.50 ± 0.00	0.66 ± 0.16	2.50 ± 0.00	0.83 ± 0.16	0.66 ± 0.16	0.50 ± 0.00	1.33 ± 0.60

Data is represented as mean ± SEM, (*n* = 3)

Keys:

*P. m Protius mirabilis, S. a Streptococcus aureus, E. c Escherichia coli, B. c Bacillus cereus, S. t Salmonella typhi, K. p Klebsiella pneumonia, P. a Pseudomonas aeruginosa, E. f Enterococcus faecalis, Ir.Cr* Crude methanolic extract, *Ir.Hex n*-hexane fraction, *Ir.*Cf Chloroform fraction, *Ir.EtAc* Ethyl acetate fraction, *Ir.Aq* Aqueous fraction, *Ir.Sp* Saponins, *Ir.Fl* Flavonoids

### Anthelmintic activity

All the tested fractions were effective in concentration dependent manner as shown in Table 6. The saponins fraction was observed to be most effective against *Pheretima posthuma* with an average death time of 44.00 ± 2.00, 33.67 ± 3.05 and 27.67 ± 1.53 min at concentrations of 10, 20, 40 mg/ml respectively. The anthelmintic activity observed for saponins and chloroform fractions were comparable to the positive control albendazole. The order of anthelmintic activity for various fractions of the plant against *Pheretima posthuma* was in an ascending order of saponins > chloroform > ethyl acetate > *n*-hexane > methanolic extract > aqueous fraction. Moreover, crude saponins exhibited a significant result against *Ascaridia galli* with an average death time of 29.22 ± 0.61 min at 40 mg/ml concentration. Whereas, average death time for the standard drug albendazole was 33.6 ± 1.76 at the same tested concentration.

**Table 6:** Anthelmintic activity of aerial part of *Isodon rugosus* against *Pheretima posthuma* and *Ascaridia galli*

Samples	Concentration (mg/ml)	*Pheretima posthuma*		*Ascaridia galli*	
		Paralysis time in Minutes (mean ± SD)	Death time in Minutes (mean ± SD)	Paralysis time in Minutes (mean ± SD)	Death time in Minutes (mean ± SD)
Ir.Cr	10	24.33 ± 1.15^***^	67.00 ± 2.00^***^	20.25 ± 0.57^***^	69.39 ± 0.60^***^
	20	18.67 ± 1.53^**^	60.00 ± 2.00^***^	17.01 ± 0.46^***^	61.18 ± 0.67^***^
	40	11.33 ± 1.56^ns^	40.67 ± 1.53^***^	13.48 ± 0.52^***^	53.19 ± 0.36^***^
Ir.Hex	10	23.33 ± 2.08^***^	67.33 ± 2.08^***^	28.89 ± 0.52^***^	87.34 ± 0.78^***^
	20	17.00 ± 1.00^*^	62.33 ± 1.53^***^	17.32 ± 0.96^***^	71.36 ± 0.56^***^
	40	11.67 ± 1.53^ns^	49.00 ± 1.00^***^	3.43 ± 0.67^**^	56.63 ± 0.38^***^
Ir.Cf	10	14.00 ± 1.00^ns^	50.00 ± 1.00^ns^	14.53 ± 0.67^ns^	53.01 ± 0.44^***^
	20	10.00 ± 2.00^ns^	37.67 ± 1.53^ns^	11.95 ± 0.34^ns^	41.77 ± 0.40^ns^
	40	7.33 ± 1.53^ns^	30.67 ± 2.52^ns^	9.85 ± 0.50^ns^	34.39 ± 0.55^ns^
Ir.EtAc	10	18.00 ± 2.00^ns^	52.00 ± 2.00^ns^	18.13 ± 0.49^***^	58.37 ± 1.16^***^
	20	13.00 ± 2.00^ns^	45.00 ± 2.00^*^	13.63 ± 0.40^**^	49.31 ± 0.82^***^
	40	10.00 ± 2.00^ns^	38.67 ± 1.53^**^	10.29 ± 0.56^*^	42.25 ± 0.89^***^
Ir.Aq	10	21.00 ± 2.00^**^	61.00 ± 2.00^***^	19.66 ± 0.88_***_	68.54 ± 0.81^***^
	20	19.00 ± 2.00^**^	56.00 ± 2.00^***^	15.4 ± 0.61^***^	57.15 ± 0.85^***^
	40	15.00 ± 2.00^**^	47.67 ± 1.53^***^	13.40 ± 0.69^***^	52.85 ± 0.50^***^
Ir.Sp	10	13.33 ± 1.53^ns^	44.00 ± 2.00^ns^	8.40 ± 0.9^***^	43.00 ± 1.00^**^
	20	9.00 ± 2.00^ns^	33.67 ± 3.05^ns^	8.33 ± 0.58^ns^	32.79 ± 0.54^***^
	40	5.67 ± 1.53^ns^	27.67 ± 1.53^ns^	4.33 ± 0.58^ns^	29.22 ± 0.61^**^
Albendazole	10	12.33 ± 2.08	48.67 ± 1.53	13.10 ± 1.85	47.40 ± 1.50
	20	10.33 ± 2.57	37.33 ± 1.53	9.80 ± 1.60	40.20 ± 2.25
	40	7.00 ± 2.00	28.67 ± 1.53	7.10 ± 1.01	33.60 ± 1.76
Negative control	-----	-----	-----	-----	------

Values significantly different (*: *P* < 0.05, ** *P* < 0.01 and *** *P* < 0.001) as compared to positive control drug. ns: Non significantly different in comparion to control group

Keys:

*Ir.Cr* Crude methanolic extract, *Ir.Hex n*-hexane fraction, *Ir.*Cf Chloroform fraction, *Ir.EtAc* Ethyl acetate fraction, *Ir.Aq* Aqueous fraction, *Ir.Sp* Saponins.

### Insecticidal activity

**Fig. 4:**
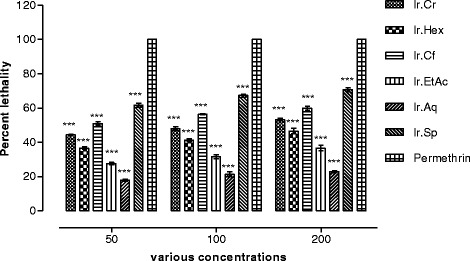
Percent lethality of various samples of *Isodon rugosus* against *Rhyzopertha dominica*

**Fig. 5:**
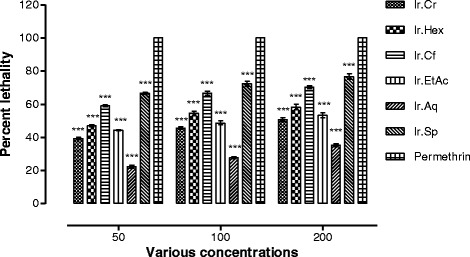
Percent lethality of various samples of *Isodon rugosus* against *Tribolium castaneum*

In finding out the insecticidal potentials of different fractions, saponins caused mortality to a significant level, i.e. 70.67 ± 1.20 and 76.65 ± 1.65% mortality against *R. dominica* and *T. castaneum* respectively at a concentration of 200 mg/ml, while at a concentration of 50 mg/ml saponins exhibited 61.66 ± 1.20 and 66.67 ± 0.67% mortality against *R. dominica* and *T. castaneum* respectively. Among other fractions, chloroform was more effective against *R. dominica* and *T. castaneum* showing 60.00 ± 1.15 and 70.33 ± 0.88% mortality against *R. dominica* and *T. castaneum* respectively at concentration of 200 mg/ml, while showed 51.00 ± 1.15 and 59.00 ± 0.57% mortality against *R. dominica* and *T. castaneum* respectively at a concentration of 50 mg/ml. The methanolic extract and *n-*hexane fraction also showed moderate insecticidal effects against both the insects as shown in Figs. 4 and 5.

### Anti-termite activity

The anti-termite activity of various samples of *I. rugosus* showed prominent lethality against termites. The highest activity was attributed to saponins, killing almost all the termites on the first day as shown in Table 7. The second highest activity was shown by ethyl acetate fraction which demonstrated 93.00 ± 0.57% lethality on the first day and 100% lethality on the second day. Other fractions including chloroform, *n*-hexane and aqueous fractions showed moderate activity causing 77.32 ± 1.32, 50.33 ± 0.88 and 48.33 ± 0.88% lethality respectively on the first day. On the third day almost all the insects were killed by all the plant’s samples.

**Table 7:** Anti-termite effect of various samples of *Isodon rugosus*

Samples	Number of Termites treated	Days	Percent lethality (mean ± SEM)
Ir.Cr	25	1	45.00 ± 1.15^***^
		2	77.32 ± 1.32^***^
		3	100.0 ± 0.00^ns^
Ir.Hex	25	1	50.33 ± 0.88^***^
		2	69.32 ± 1.32^***^
		3	100.0 ± 0.00^ns^
Ir.Cf	25	1	77.32 ± 1.32^***^
		2	96.67 ± 1.20^ns^
		3	100.0 ± 0.00^ns^
Ir.EtAc	25	1	93.00 ± 0.57^ns^
		2	100.0 ± 0.00^ns^
		3	100.0 ± 0.00^ns^
Ir.Aq	25	1	48.33 ± 0.88^***^
		2	74.00 ± 0.57^***^
		3	100.0 ± 0.00^ns^
Ir.Sp	25	1	100.0 ± 0.00^ns^
		2	100.0 ± 0.00^ns^
		3	100.0 ± 0.00^ns^
Permethrin	25	1 2 3	100.0 ± 0.00100.0 ± 0.00100.0 ± 0.00

Data is represented as mean ± SEM; (*n* = 3). Values significantly different (*** *P* < 0.001) as compared to the control drug

Keys:

*Ir.Cr* Crude methanolic extract, *Ir.Hex n*-hexane fraction, *Ir.*Cf Chloroform fraction, *Ir.EtAc* Ethyl acetate fraction, *Ir.Aq* Aqueous fraction, *Ir.Sp* Saponins

### Anti-Pharaoh (ants) activity

The determination of anti-Pharaoh ants potentials of *I. rugosus* reveal that among all the plant’s samples, saponins showed 100% activity against Pharaoh at all the tested concentrations except at 0.5 mg/ml which was 96.33 ± 0.88%. The second highest activity can be attributed to the ethyl acetate fractions of *I. rugosus* which displayed 72.00 ± 1.15, 77.67 ± 0.67, 80.33 ± 0.33, 86.33 ± 0.88, 90.67 ± 0.67 and 100.0 ± 0.57% lethality at concentrations of 0.5, 1, 2, 4, 6 and 8 mg/ml respectively. The LD_50_ shown by saponins, ethyl acetate and chloroform fractions were smaller than 0.1 mg/ml, whereas LD_50_ for methanolic extract, *n*-hexane and aqueous fractions were 0.85, 1.13 and 3.37 mg/ml respectively as shown in Table 8.

**Table 8:** Anti-Pharaoh ant effect of various samples of *Isodon rugosus*

Samples	Dose (mg/ml)	Total treated	No. repeated	Mortality(%) mean ± SEM	LD_50_ (mg/ml)
Ir.Cr	0.5	20	3	41.33 ± 0.88^***^	0.85
	1	20		52.00 ± 0.57^***^	
	2	20		57.67 ± 0.67^***^	
	4	20		58.00 ± 0.57^***^	
	6	20		70.00 ± 0.00^***^	
	8	20		81.33 ± 0.88^***^	
Ir.Hex	0.5	20	3	39.67 ± 1.20^***^	1.13
	1	20		49.00 ± 1.15^***^	
	2	20		53.00 ± 0.57^***^	
	4	20		56.67 ± 0.67^***^	
	6	20		58.00 ± 0.57^***^	
	8	20		80.67 ± 1.20^***^	
Ir.Cf	0.5	20	3	67.33 ± 1.45^***^	<0.1
	1	20		68.67 ± 0.67^***^	
	2	20		73.33 ± 0.88^***^	
	4	20		76.00 ± 1.15^***^	
	6	20		85.00 ± 0.57^**^	
	8	20		95.33 ± 0.88^ns^	
Ir.EtAc	0.5	20	3	72.00 ± 1.15 ^***^	<0.1
	1	20		77.67 ± 0.67 ^***^	
	2	20		80.33 ± 0.33^**^	
	4	20		86.33 ± 0.88^*^	
	6	20		90.67 ± 0.67^ns^	
	8	20		100.0 ± 0.57^ns^	
Ir.Aq	0.5	20	3	29.00 ± 1.15^***^	3.37
	1	20		41.33 ± 0.88 ^***^	
	2	20		44.67 ± 1.20^***^	
	4	20		53.33 ± 0.33 ^***^	
	6	20		66.00 ± 0.57^***^	
	8	20		80.67 ± 1.76^***^	
Ir.Sp	0.5	20	3	96.33 ± 0.88^ns^	<0.1
	1	20		100.0 ± 0.00^ns^	
	2	20		100.0 ± 0.00^ns^	
	4	20		100.0 ± 0.00^ns^	
	6	20		100.0 ± 0.00^ns^	
	8	20		100.0 ± 0.00^ns^	
Permithrin	0.5	20	3	93.33 ± 4.40	<0.1
	1	20		98.33 ± 1.66	
	2	20		100.0 ± 0.00	
	4	20		100.0 ± 0.00	
	6	20		100.0 ± 0.00	
	8	20		100.0 ± 0.00	
Distilled Water	Negative control	20	3	0.00	0.00

Data is represented as mean ± SEM; (*n* = 3). Values significantly different (*: *P* < 0.05, ** *P* < 0.01 and *** *P* < 0.001) as compared to the control drug

Keys:

*Ir.Cr* Crude methanolic extract, *Ir.Hex n*-hexane fraction, *Ir.*Cf Chloroform fraction, *Ir.EtAc* Ethyl acetate fraction, *Ir.Aq* Aqueous fraction, *Ir.Sp* Saponins.

## Discussion

The origin and nature of drug used against specific diseases make a sound difference in the therapy and prognosis. The efficacy and safety of synthetic and natural drugs have been investigated and compared by multiple reporters [39, 40]. Similarly, the use of drugs derived from natural sources have been found comparatively safe and have been preferred against synthetic drugs [41]. Several groups of natural compounds have been reported to possess variety of potentials, for examples, flavonoids have been shown to possess antimicrobial activity that is also obvious in our current study [42]. Similarly, parasitic worms causing infections of human beings and animals are well-known to have negative health consequences and a significant reduction in body ability to resist other diseases. To find compounds with anthelmintic activity, a variety of substances have been investigated using different species of worms like earthworms, ascaris, heterakis and nippostrongylus [42, 43]. Among all these species, earthworms have been used generally for the preliminary evaluation of anthelmintic compounds in-vitro due to physiological similarity with intestinal worms, their reaction to anthelmintics and are easily accessible [44]. It has been established that all those anthelmintics which are toxic to earthworms are regarded as anthelmintic agents. To minimize losses caused by helminths infections, commercial anthelminthics have been used for decades. However, the threats of anthelminthic resistance, poor availability, risk of residue and high cost especially to low income people in developing countries have led to the need of other alternative control methods [45]. Screening and proper evaluation of the claimed medicinal plants could offer the possible alternatives that may both be sustainable and environmentally acceptable instead of those having toxic effect [46].

Results of the current study reveal that saponins were most effective against *P. posthuma* exhibiting an average death time of 27.67 ± 1.53 min which was comparable with the standard drug albendazole with average death time of 28.67 ± 1.53 at the same concentration (40 mg/ml). Furthermore, crude saponins were most effective against *Ascaridia galli* with average death time of 29.22 ± 0.61 min at 40 mg/ml. The average death time for standard drug albendazole was 33.6 ± 1.76 at the same concentration. Researchers have proven that saponins possess excellent anthelmintic potential which has also been depicted in our investigations [36].

In the current study, we observed that saponins were also active against insects in a similar way reported earlier [47]. Moreover, the saponins have also been reported to possess anthelmintic, antitussive, anticancer, fungicidal and antiviral properties [48–51]. Actually the saponins help the plants to protect against pests and parasites [49, 52–55]. Saponins may cause inhibition of food uptake in the insects’ gut and interrupt in their digestion which lead to stop their growth [47]. They are considered as first line choice for the proper eradication of insects due to their protease inhibitory properties and interaction with cholesterol by disturbing the synthesis of moulting hormones [56]. The saponins isolated from various plants have been reported to possess toxicity profile and have been demonstrated with prominent haemolytic potential. The haemolytic potential and toxicity profile of the saponins make it inevitable that the saponins of each and every plant should be subjected for the evaluation of safety profile in regards to the human body.

The GC-MS analysis showed 57 compounds in the ethyl acetate fraction of *I. rugosus*. The bioactive compounds identified were palmitic acid, hinokiol, α-amyrin, phytol, ethyl linolate, cyclohexanone, hinokione, methyl palmitate, ethyl palmitate and stigma sterol acetate. The literature survey of the bioactive compounds reveals the importance of *I. rugosus* regarding various biological activities. The compounds identified in this plant have been reported previously with significant biological activities. Cyclohexanone possess strong antibacterial activity [57]. Red and white wine containing succinic acid have been reported with strong antibacterial activity [58]. The succinic acid derivatives have been reported to treat the infants urinary tract infection [59]. Similarly, the succinic acid have also been reported for antibacterial potential produced by yeast during fermentation [60]. Palmitic acid have been reported to possess antimicrobial potential [61, 62]. Phytol have been reported for its antibacterial [63], antimicrobial [64] and insecticidal potentials [62]. Hinokiol isolated from *Magnolia officinalis* have been used for antibacterial activity [65]. Ethyl linolate have been used for the treatment of acne vulgaris [66] and topically as antibacterial agent [67]. In the same way, stigmasterol acetate has also been used as antibacterial agent [68]. Likewise, alpha-amyrin possess strong antibacterial activity [69]. Cyclohexanone have been reported to used as a solvent and as a building block in the production of various organic compounds such as insecticidal [70]. Methyl palmitate have been reported to possess insecticidal activity [71]. Palmitic acid have been reported to possess strong anti mosquitoes activities isolated from *Acanthus montanus* [72]. Ethyl palmitate is larvicidal and insecticidal agent and has been used in insecticidal sprays [73]. Similarly, hinokione isolated from *Sabina vulgaris* have been reported to possess insecticidal potential [74]. Stigmasterol acetate has mosquito repellent property [75]. Alpha-Amyrin have been reported for insecticidal activity [69].

## Conclusions

It can be concluded that *Isodon rugosus* possess strong antibacterial, insecticidal and anthelmintic potentials. The gas chromatography-mass spectroscopy analysis also elucidated a collection of biologically important compounds putatively known for their antibacterial, insecticidal and anthelmintic potentials. Based on the experimental analysis, it may also be deduced that *Isodon rugosus* is a good source of antibacterial, insecticidal and anthelmintic compounds. The overwhelming role of ethyl acetate fraction in various biological assays and the GC-MS analysis confirm that this fraction might be an appropriate target for the isolation of bioactive compounds.

## Abbreviations

B. c: Bacillus cereus
*E. c*: *Escherichia coli*
*E. f*: *Enterococcus faecalis*
GC-MS: gas chromatography-mass spectrometry
Ir.Aq: aqueous fraction
Ir.Cf: chloroform fraction
Ir.Cr: Crude methanolic extract
Ir.EtAc: ethyl acetate fraction
Ir.Fl: flavonoids
Ir.Hex: *n*-hexane fraction
Ir.Sp: saponins
*K. p*: *Klebsiella pneumonia*
LD_50_: median lethal dose
MDR: multi drug resistant
MIC: minimum inhibitory concentration
NCCLS: National Committee for Clinical Laboratory Standards
*P. a*: *Pseudomonas aeruginosa.*
*P. m*: *Protius mirabilis*
*S. a*: *Streptococcus aureus*
*S. t*: *Salmonella typhi*
SEM: standard error mean
ZOI: zone of inhibition

## References

[1] Noumedem JAK, Mihasan M, Lacmata ST (2013). Antibacterial activities of the methanol extracts of ten Cameroonian vegetables against gram-negative multidrug-resistant bacteria. BMC Complement Altern Med.

[2] Okeke IN, Laxminarayan R, Bhutta ZA, Duse AG, Jenkins P, O'Brien TF, et al. Antimicrobial resistance in developing countries. Part I: recent trends and current status. Lancet Infect Dis. 2005;5(8):481–93. 10.1016/S1473-3099(05)70189-4 16048717

[3] Ayaz M, Subhan F, Sadiq A, Ullah F, Ahmed J, Sewell R (2017). Cellular efflux transporters and the potential role of natural products in combating efflux mediated drug resistance. Front Biosci.

[4] Ayaz M, Subhan F, Ahmed J, Khan A-U, Ullah F, Sadiq A, et al. Citalopram and Venlafaxine differentially augments antimicrobial properties of antibiotics. Acta Poloniae Pharmaceutica ñ Drug Research. 2015;72(6):1269–78.

[5] Bandow JE, Brötz H, Leichert LIO, Labischinski H, Hecker M (2003). Proteomic approach to understanding antibiotic action. Antimicrob Agents Chemother.

[6] Ullah F, Malik SA, Ahmed J, Ullah F, Shah SM, Ayaz M, et al. Investigation of the genetic basis of tetracycline resistance in *Staphylococcus aureus* from Pakistan. Trop J Pharm Res. 2012;11(6):925–31.

[7] Khalafi-Nezhad A, Rad M, Mohabatkar H, Asrari Z, Hemmateenejad B (2005). Design, synthesis, antibacterial and QSAR studies of benzimidazole and imidazole chloroaryloxyalkyl derivatives. Bioorg Med Chem.

[8] Clardy J, Walsh C (2004). Lessons from natural molecules. Nature.

[9] Ullah F, Ayaz M, Sadiq A, Hussain A, Ahmad S, Imran M, et al. Phenolic, flavonoid contents, anticholinesterase and antioxidant evaluation of *Iris germanica* var; florentina. Nat Prod Res. 2016;30(12):1440–4. 10.1080/14786419.2015.1057585 26166432

[10] Dhar D, Sharma R, Bansal G (1982). Gastro-intestinal nematodes in sheep in Kashmir. Vet Parasitol.

[11] Blumenthal DS, Schultz MG (1975). Incidence of intestinal obstruction in children infected with Ascaris Lumbricoides. AmJTrop Med Hyg.

[12] Tagboto S, Townson S (2001). Antiparasitic properties of medicinal and other naturally occurring products. Adv Parasitol.

[13] Chiejina S. The epidemiology of helminth infections of domesticated animals in the tropics with emphasis on fascioliasis and parasitic gastroenteritis. Perspect Helminthol. 2001:41–87.

[14] Liu LX, Weller PF (1996). Antiparasitic drugs. N Engl J Med.

[15] Calabrese EJ, Baldwin LA (2003). Toxicology rethinks its central belief. Nature.

[16] Duke SO, Baerson SR, Dayan FE, Rimando AM, Scheffler BE, Tellez MR, et al. United States Department of agriculture–agricultural research service research on natural products for pest management. Pest Manag Sci. 2003;59(6–7):708–17. 10.1002/ps.633 12846321

[17] Ayaz M, Junaid M, Ullah F, Sadiq A, Ovais M, Ahmad W, Ahmad S, Zeb A: Chemical profiling, antimicrobial and isecticidal evaluations of Polygonum hydropiper L. BMC Complement Altern Med. 2016;16:502. 10.1186/s12906-016-1491-4 PMC5139080 27919287

[18] Harris ES (2011). Not-so-traditional Chinese medicine: the example of Donglingcao (*Isodon rubescens*). Arnoldia.

[19] Janbaz KH, Arif J, Saqib F, Imran I, Ashraf M, Zia-Ul-Haq M, et al. In-vitro and in-vivo validation of ethnopharmacological uses of methanol extract of *Isodon rugosus* Wall. Ex Benth.(Lamiaceae). BMC Complement Altern Med. 2014;14(1):71. 10.1186/1472-6882-14-71 PMC3974051 24559094

[20] Haq F, Ahmad H, Ullah R, Iqbal Z (2012). Species diversity and ethno botanical classes of the Flora of Allai Valley district Battagram Pakistan. Int J Plant Res.

[21] Ahmad M, Sultana S, Fazl-i-Hadi S, Ben Hadda T, Rashid S, Zafar M, et al. An Ethnobotanical study of medicinal plants in high mountainous region of Chail valley (district swat-Pakistan). J Ethnobiol Ethnomed. 2014;10(1):1. 10.1186/1746-4269-10-36 PMC4022037 24739524

[22] Sabeen M, Ahmad SS (2009). Exploring the folk medicinal flora of Abbotabad city Pakistan. Ethnobotanical Leaflets.

[23] Zeb A, Ahmad S, Ullah F, Ayaz M, Sadiq A: Anti-nociceptive activity of ethnomedicinally important analgesic plant Isodon rugosus Wall. ex Benth: Mechanistic study and identifications of bioactive compounds. Front Pharmacol. 2016;7:200. 10.3389/fphar.2016.00200 PMC4933699 27458379

[24] Ayaz M, Junaid M, Ahmed J, Ullah F, Sadiq A, Ahmad S, et al. Phenolic contents, antioxidant and anticholinesterase potentials of crude extract, subsequent fractions and crude saponins from *Polygonum hydropiper* L. BMC Complement Altern Med. 2014;14(1):145. 10.1186/1472-6882-14-145 PMC4018186 24884823

[25] Ahmad S, Ullah F, Ayaz M, Zeb A, Ullah F, Sadiq A (2016). Antitumor and anti-angiogenic potentials of isolated crude saponins and various fractions of *Rumex hastatus* D. Don. Biol Res.

[26] Zeb A, Sadiq A, Ullah F, Ahmad S, Ayaz M (2014). Phytochemical and toxicological investigations of crude methanolic extracts, subsequent fractions and crude saponins of *Isodon rugosus*. Biol Res.

[27] Ayaz M, Junaid M, Ullah F, Sadiq A, Subhan F, Khan MA, et al. Molecularly characterized solvent extracts and saponins from *Polygonum hydropiper* L show high anti-angiogenic, anti-tumor, brine shrimp and fibroblast NIH/3T3 cell line cytotoxicity. Front Pharmacol. 2016;7:74. 10.3389/fphar.2016.00074 PMC4814464 27065865

[28] Zeb A, Sadiq A, Ullah F, Ahmad S, Ayaz M (2014). Investigations of anticholinestrase and antioxidant potentials of methanolic extract, subsequent fractions, crude saponins and flavonoids isolated from *Isodon rugosus*. Biol Res.

[29] Ayaz M, Junaid M, Ullah F, Sadiq A, Khan MA, Ahmad W, et al. Comparative chemical profiling, cholinesterase inhibitions and anti-radicals properties of essential oils from *Polygonum hydropiper* L: a preliminary anti-Alzheimer's study. Lipids Health Dis. 2015;14(1):141. 10.1186/s12944-015-0145-8 PMC4632677 26530857

[30] Adams R (2007). Identification of essential oil components by gas chromatography/mass spectrometry.

[31] Ayaz M, Subhan F, Ahmed J, Khan A-U, Ullah F, Ullah I, et al. Sertraline enhances the activity of antimicrobial agents against pathogens of clinical relevance. J Biol Res-Thessaloniki. 2015;22(1):4. 10.1186/s40709-015-0028-1 PMC4449573 26029671

[32] Sadiq A, Ahmad S, Ali R, Ahmad F, Ahmad S, Zeb A, et al. Antibacterial and antifungal potentials of the solvents extracts from *Eryngium caeruleum, Notholirion thomsonianum and Allium consanguineum*. BMC Complement Altern Med. 2016;16:478. 10.1186/s12906-016-1465-6 PMC5122145 27881119

[33] Shah SM, Ayaz M, Khan A-U, Ullah F, Farhan, Shah A-U-HA, Iqbal H, Hussain S: 1,1-Diphenyl,2-picrylhydrazyl free radical scavenging, bactericidal, fungicidal and leishmanicidal properties of Teucrium stocksianum. Toxicology and Industrial Health 2015, 31(11):1037–1043. 10.1177/0748233713487250 23625908

[34] National Committee for Clinical Laboratory Standards: 1993 Mfdastfbtga, 3rd ed., Approved standard M7-A3. NCCLS, Villanova, PA.

[35] Vigar Z (1984). Atlas of medical parasitology.

[36] Ayaz M, Junaid M, Subhan F, Ullah F, Sadiq A, Ahmad S, et al. Heavy metals analysis, phytochemical, phytotoxic and anthelmintic investigations of crude methanolic extract, subsequent fractions and crude saponins from *Polygonum hydropiper* L. BMC Complement Altern Med. 2014;14(1):465. 10.1186/1472-6882-14-465 PMC4289404 25472835

[37] Ahn Y, Kim G, Cho K: Bioassay system for insecticidal compounds. In: Proceedings of the third symposium on the biochemical methodology for the research and development of the bioactive substances, held at Seoul, Republic of Korea*:* 1995; 1995: 495–506.

[38] Salihah Z, Khatoon R, Khan A, Alamzeb SA (1993). A termite trap, NIFATERMAP, for capturing large number of field population of Heterotermes Indicola. Proc Pakistan Cong Zool.

[39] Ullah I, Subhan F, Ayaz M, Shah R, Ali G, Haq IU, et al. Anti-emetic mechanisms of *Zingiber officinale* against cisplatin induced emesis in the pigeon; behavioral and neurochemical correlates. BMC Complement Altern Med. 2015;15(1):34. 10.1186/s12906-015-0556-0 PMC4355376 25888212

[40] Sadiq A, Mahmood F, Ullah F, Ayaz M, Ahmad S, Haq FU, et al. Synthesis, anticholinesterase and antioxidant potentials of ketoesters derivatives of succinimides: a possible role in the management of Alzheimer's. Chem Central J. 2015;9(1):31. 10.1186/s13065-015-0107-2 PMC4461796 26064188

[41] Kahl R, Kappus H (1993). Toxicology of the synthetic antioxidants BHA and BHT in comparison with the natural antioxidant vitamin E. Zeitschrift für Le0bensmittel-Untersuchung und-Forschung.

[42] Cushnie T, Lamb AJ (2005). Antimicrobial activity of flavonoids. Int J Antimicrob Agents.

[43] Theodorides V, Gyurik R, Kingsbury W, Parish R (1976). Anthelmintic activity of albendazole against liver flukes, tapeworms, lung and gastrointestinal roundworms. Cell Mol Life Sci.

[44] Viigar Z (1984). Atlas of medical Parasitology.

[45] Waller PJ (1997). Sustainable helminth control of ruminants in developing countries. Vet Parasitol.

[46] Shah S, Shah SMM, Ahmad Z, Yaseen M, Shah R, Sadiq A, Khan S, Khan B. Phytochemicals, in vitro antioxidant, total phenolic contents and phytotoxic activity of Cornus macrophylla Wall bark collected from the north-west of Pakistan. Pak J Pharm Sci. 2015;28(1):23-8. 25553682

[47] Adel MM, Sehnal F, Jurzysta M (2000). Effects of alfalfa saponins on the moth Spodoptera Littoralis. J Chem Ecol.

[48] Wang G-X, Han J, Zhao L-W, Jiang D-X, Liu Y-T, Liu X-L (2010). Anthelmintic activity of steroidal saponins from< i> Paris polyphylla</i>. Phytomedicine.

[49] Yan L, Zhang Y, Gao W, Man S, Wang Y (2009). In vitro and in vivo anticancer activity of steroid saponins of Paris polyphylla var. yunnanensis. Exp Oncol.

[50] Marston A, Gafner F, Dossaji S, Hostettmann K (1988). Fungicidal and molluscicidal saponins from< i> Dolichos kilimandscharicus</i>. Phytochemistry.

[51] Amoros M, Fauconnier B, Girre R (1987). In vitro antiviral activity of a saponin from< i> Anagallis Arvensis</i>, Primulaceae, against herpes simplex virus and poliovirus. Antivir Res.

[52] Milgate J, Roberts D (1995). The nutritional & biological significance of saponins. Nutr Res.

[53] Oleszek W, Hoagland R, Zablotowicz R. Ecological significance of plant saponins. Principles and Practices in Plant Ecology: Allelochemical Interactions CRC Press, New York; 1999. p. 451–65.

[54] Potter DA, Kimmerer TW (1989). Inhibition of herbivory on young holly leaves: evidence for the defensive role of saponins. Oecologia.

[55] Shah SMM, Sadiq A, Shah SMH, Khan S: Extraction of saponins and toxicological profile of Teucrium stocksianum boiss extracts collected from District Swat, Pakistan. Biol Res. 2014;47(1):65. 10.1186/0717-6287-47-65 PMC4271446 25730474

[56] Harmatha J, Dinan L. Interaction of dimeric ecdysteroids, glycosidic ecdysteroid conjugates and ecdysis-disturbing saponins with the ecdysteroid receptor assessed by means of the *Drosophila melanogaster* B-II bioassay. Arthropods: Chemical, Physiological and Environmental Aspects. 2002:79–84.

[57] Ovsyannikova M, Vol’eva V, Belostotskaya I, Komissarova N, Malkova A, Kurkovskaya L (2013). Antibacterial activity of substituted 1, 3-Dioxolanes. Pharm Chem J.

[58] Daglia M, Papetti A, Grisoli P, Aceti C, Dacarro C, Gazzani G (2007). Antibacterial activity of red and white wine against oral streptococci. J Agric Food Chem.

[59] Hansson S, Dhamey M, Sigström O, Sixt R, Stokland E, Wennerström M, et al. Dimercapto-succinic acid scintigraphy instead of voiding cystourethrography for infants with urinary tract infection. J Urol. 2004;172(3):1071–4. 10.1097/01.ju.0000135337.71154.60 15311040

[60] Basso L, Alves D, Amorim H (1997). The antibacterial action of succinic acid produced by yeast during fermentation. Rev Microbiol.

[61] Yff BT, Lindsey KL, Taylor MB, Erasmus DG, Jäger AK (2002). The pharmacological screening of Pentanisia prunelloides and the isolation of the antibacterial compound palmitic acid. J Ethnopharmacol.

[62] Senthilkumar N, Murugesan S, Vijayalakshmi K (2012). GC–MS–MS analysis of Trichili a connaroides (Wight & Arn.) Bentv (Meliaceae): a tree of ethnobotanical records. Asian J Plant Sci Res.

[63] Inoue Y, Hada T, Shiraishi A, Hirose K, Hamashima H, Kobayashi S (2005). Biphasic effects of geranylgeraniol, teprenone, and phytol on the growth of *Staphylococcus aureus*. Antimicrob Agents Chemother.

[64] Padmini E, Valarmathi A, Rani Usha M (2010). Comparative analysis of chemical composition and antibacterial activities of *Mentha spicata* and *Camellia sinensis*. Asian J Exp Biol Sci.

[65] Ho KY, Tsai CC, Chen CP, Huang JS, Lin CC (2001). Antimicrobial activity of honokiol and magnolol isolated from Magnolia Officinalis. Phytother Res.

[66] Charakida A, Charakida M, Chu A (2007). Double-blind, randomized, placebo-controlled study of a lotion containing triethyl citrate and ethyl linoleate in the treatment of acne vulgaris. Br J Dermatol.

[67] Jelenko C, Wheeler M, Anderson A, Callaway B, McKinley J (1975). Studies in burns: XIV, Heling in burn wounds treated with ethyl Linoleate alone or in combination with selected topical antibacterial agents. Ann Surg.

[68] Sharma RK (1993). Phytosterols: wide-spectrum antibacterial agents. Bioorg Chem.

[69] Devi S, Joseph J, Rajkumar J (2015). In silico approach of antibacterial compounds from mangrove-Avicennia Marina through docking analysis. Biomed Res.

[70] Sivaraj C, Reddy BM, Rao PK (1988). Selective dehydrogenation of cyclohexanol to cyclohexanone on cu ZnO al 2 O 3 catalysts. Appl Catal.

[71] Wang Y, Wang H, Jin Y, Bu C, Cheng J, Zhao L, et al. Assessment of the contact toxicity of methyl palmitate on Tetranychus viennensis (Acari: Tetranychidae). J Econ Entomol. 2010;103(4):1372–7. 10.1603/ec09128 20857750

[72] Amin E, Radwan MM, El-Hawary SS, Fathy MM, Mohammed R, Becnel JJ, et al. Potent insecticidal secondary metabolites from the medicinal plant *Acanthus montanus*. Rec Nat Prod. 2012;6:301–5.

[73] Tisdale WH, Bake LS (1936). Combined fungicidal and insecticidal spray materials. Google patents.

[74] Yan H, Feng R, Chen L, Chen A, Li G, Zhang X (2007). Isolation, identification and insecticidal activities of six terpenoids in *Sabina vulgaris*.

[75] Chogo J, Crank G (1981). Chemical composition and biological activity of the Tanzanian plant Ocimum Suave. J Nat Prod.

